# Failure mechanisms and stability control of roadways in thermally damaged rock under asymmetric stress: a case study

**DOI:** 10.1038/s41598-026-45210-z

**Published:** 2026-04-11

**Authors:** Zhiqiang Wang, Lu Lin, Jingkai Li, Binyu Liu, Xinyu An, Peng Wang

**Affiliations:** 1 China University of Mining and Technology-Beijing, Beijing, 100083 China; 2 China-Russia Dynamics Research Center, China University of Mining and Technology-Beijing, Beijing, 100083 China; 3 Key Laboratory of Xinjiang Coal Resources Green Mining, Ministry of Education, Xinjiang Institute of Engineering, Urumqi, 830023 China; 4 School of Mining and Geomatics Engineering, Hebei University of Engineering, Handan, 056038 China

**Keywords:** Thermally damaged rock, Surrounding rock control, Asymmetric stress, Failure mechanism, Synergistic support, Engineering, Natural hazards, Solid Earth sciences

## Abstract

Stability maintenance of deep roadways in thermally damaged rock masses presents complex challenges under asymmetric stress conditions. This study investigates the failure mechanisms and proposes a control strategy using an integrated methodology of laboratory testing, theoretical analysis, and field validation. Triaxial compression tests with Acoustic Emission monitoring indicated that the mechanical degradation of thermally damaged rock is primarily influenced by the propagation of pre-existing microcracks. An analytical model based on the complex variable method quantified the stress field around a rectangular opening, incorporating the effect of principal stress rotation. The analysis revealed a coupled failure mechanism where stress asymmetry governs the inclined X-shaped plastic failure geometry while degraded rock properties determine the extent of the plastic zone. To address this mechanism, a Reinforcement-Anchorage-Confinement (RAC) collaborative support system was developed. Numerical simulations and a field application demonstrated that the RAC system controls fracture propagation and maintains roadway deformation within acceptable operational limits. This research provides a mechanistic framework for roadway stability control in similar geomechanical environments.

## Introduction

The stability of underground excavations in rock masses subjected to high-temperature alteration presents complex challenges in engineering fields such as underground coal mining, geothermal energy extraction, and deep mining. Thermal exposure transforms intact rock into a fractured medium characterized by reduced strength and stiffness. A prominent example of this phenomenon is the formation of thermally altered zones resulting from subsurface coal fires^1^. Understanding the failure mechanisms within these thermally damaged rock masses is fundamental for developing effective control strategies.

This geomechanical challenge is particularly acute in underground coal mining, an industry characterized by its immense scale. For instance, in China alone, the annual excavation length of new roadways exceeds 12,000 km^2–4^. Conventional support systems frequently fail in thermally damaged rock masses, leading to safety risks and economic losses. This problem is compounded by the widespread use of rectangular roadways, which are prevalent due to their high space utilization and compatibility with standard excavation methods^5–8^. However, the angular corners of these roadways induce high stress concentrations. While manageable in competent rock, these concentrations become critical initiation points for failure in weak, thermally damaged materials^9–11^. Consequently, roadway stability is governed by the interaction between material deterioration and geometric stress concentrations. An accurate prediction of the stress distribution and the resulting plastic zone is essential for rational support design. However, this poses an analytical challenge, as conventional elastoplastic theories have limitations in modeling non-circular openings under asymmetric loading and often require approximate methods that may lack sufficient accuracy^12–15^.

To overcome these analytical limitations, this study utilizes the complex variable method. This approach is well-established in rock mechanics for providing closed-form analytical solutions for stress and displacement around complex openings^15,16^. Previous studies have successfully applied this method to analyze roadways of various shapes, such as trapezoidal and super-large rectangular sections, and to consider the effects of anisotropic stress and varying lateral pressure coefficients^17–19^. However, a review of the existing literature indicates that many analytical solutions are based on a coaxial stress field, where the principal stresses are aligned with the roadway axes. In complex geological conditions, such as those influenced by adjacent mining, tectonic stress, or thermal coupling, principal stress rotation is common and can be significant. Existing coaxial models cannot capture the asymmetric stress concentrations that arise under rotated principal stress fields; they systematically misidentify both the location and the magnitude of the peak stress concentration, leading to symmetric support designs that are insufficient for asymmetric failure conditions. This study addresses this problem by incorporating the principal stress rotation angle *β* explicitly into the boundary conditions of the complex variable solution for a rectangular opening.

In addition to elastic stress analysis, an accurate understanding of the plastic zone is essential for designing effective support systems. Foundational theories for plastic zone calculation in circular tunnels^20,21^ have been continuously refined to incorporate various factors^22–25^ and extended to more complex conditions, including tunnel interactions and three-dimensional stress states^26–28^. For example, the concept of butterfly-shaped plastic zones has been introduced to describe failure zones around circular roadways under non-uniform stress, underscoring the governing role of stress asymmetry^28–30^.

Despite advances in roadway stability analysis, characterizing the coupled mechanical response of a thermally damaged rock mass to the in-situ stress field remains limited. Conventional supports developed for competent rock perform poorly in thermally damaged conditions. Direct application of empirical designs increases the risk of instability. These observations indicate the need for a design framework based on the specific failure mechanisms of thermally damaged rock.

This paper details an investigation that integrates theoretical analysis, laboratory testing, numerical validation, and field implementation, focusing on a case from the Wangcaihuopan Mine. An analytical solution for the stress field around a rectangular roadway is established using the complex variable method, incorporating the effect of principal stress rotation. This model is validated against laboratory test results and numerical simulations. It is then used to analyze how the interaction between stress asymmetry and rock degradation governs roadway failure patterns. To address this mechanism, a synergistic support strategy, termed the Reinforcement-Anchorage-Confinement (RAC) system, was developed. Numerical simulations and a field application demonstrated that the RAC system controls fracture propagation and maintains roadway deformation within permissible operational limits. This research provides a mechanistic insights and a field-validated solution for roadway stability in similar geomechanical environments.

## Geomechanical conditions and rock mass characterization

### Engineering background and observed failure modes

**Fig. 1:**
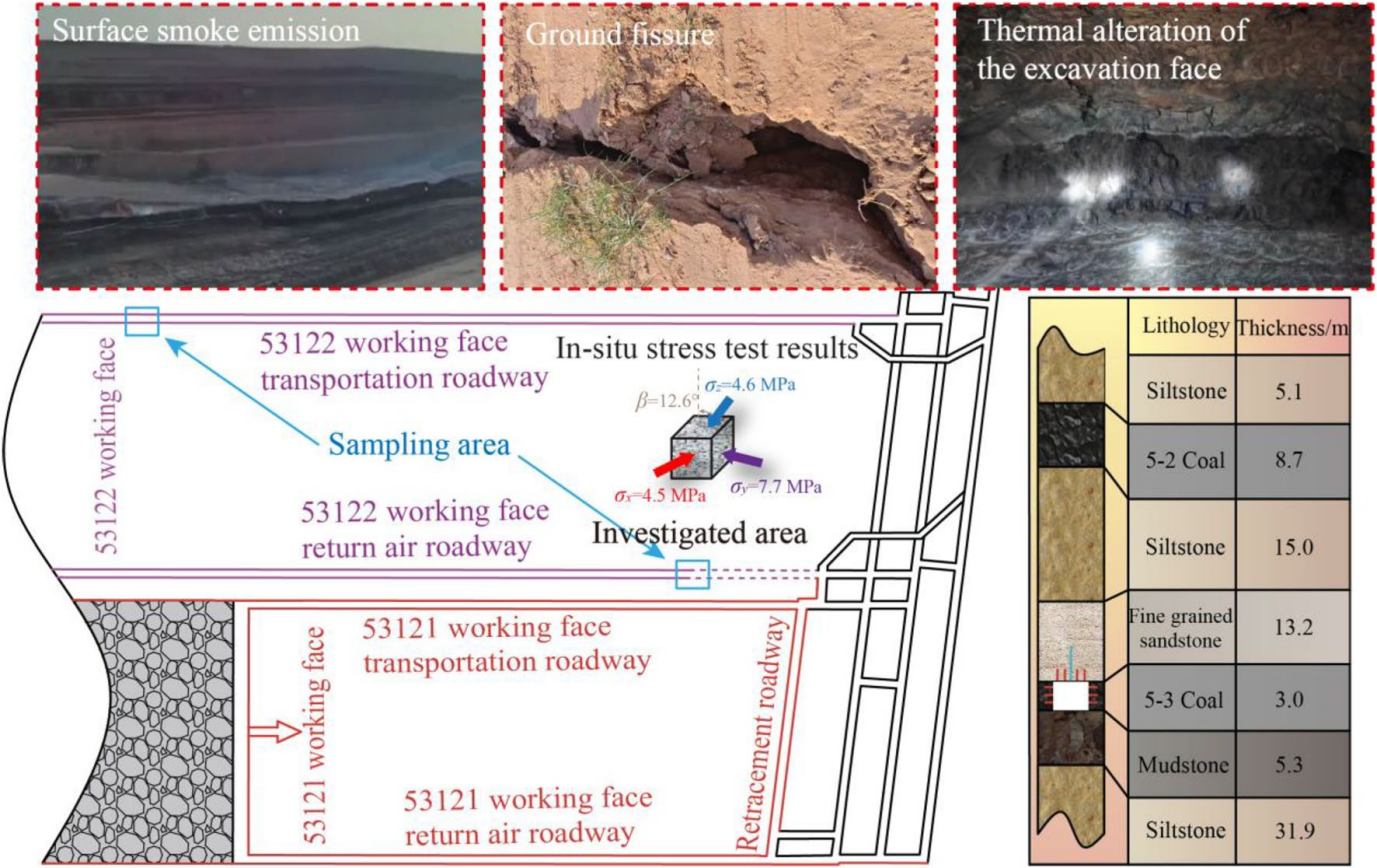
Geological context and layout of the investigated roadway in the mine.

The study area is located at the Wangcaihuopan Mine within the Shenfu Mining Area. Subsurface coal fires in the outcropping No. 5-2 coal seam have created extensive ground fissures and surface smoke emissions, indicating active and prolonged combustion. Prolonged heating has induced mineralogical transformations and fabric degradation in the overlying strata, forming a weak, fractured zone commonly referred to as burnt rock. This thermal alteration extends downward to the underlying No. 5-3 coal seam, located 15-40 m below, where mining operations are conducted. This study focuses on the 53122 working face roadway, excavated in the No. 5-3 seam at an average depth of 238 m. The roadway features a rectangular cross-section of 5.5 m (width) × 3.0 m (height). The layout of the working face and the in-situ conditions are illustrated in Fig. 1.

Field measurements of the in-situ stress state were conducted, revealing a highly anisotropic and non-coaxial stress environment. The measured principal stresses are *σ*_y_ = 7.7 MPa , *σ*_x_ = 4.5 MPa, and *σ*_z_ = 4.6 MPa. Critically, the principal stress plane in the roadway’s cross-section is rotated, with the minor principal stress axis showing a deviation of 12.6° from the vertical *Z*-axis. This rotation primarily reflects the influence of regional geological structures and topographic conditions, as the measurement was conducted prior to mining operations in the study area. It is acknowledged that subsequent mining activities, including the extraction of the adjacent 53121 working face and the formation of the associated goaf, may further perturb the local stress field and contribute to additional rotation of the principal stress orientation. The *β* = 12.6° adopted in this study therefore captures the combined effect of all contributing geological factors at the time of measurement, and represents a key external factor inducing asymmetric loading on the rectangular roadway.

**Fig. 2:**
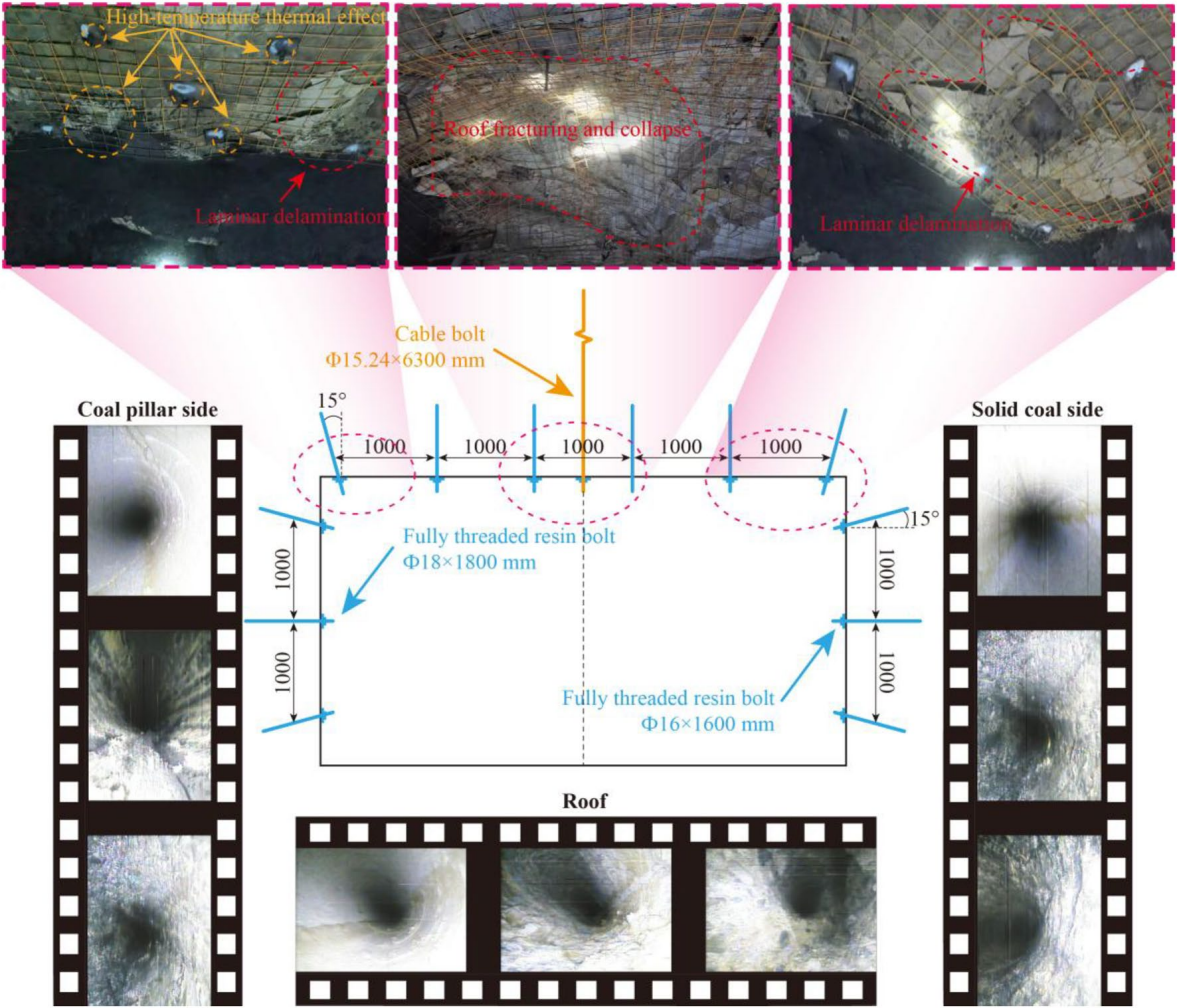
Failure modes observed in the roadway and original support paramters.

The significant thermal alteration of the surrounding rock mass is evidenced by several field observations. The excavation face exhibited extensive reddish-brown discoloration (Fig. 1), and oxidation discoloration was observed on steel bolt plates within the roadway (Fig. 2), both providing direct visual evidence of prolonged high-temperature exposure during the coal fire event. The thermal damage mechanism operates through two primary pathways: prolonged high-temperature exposure drives dehydration and mineralogical transformation of mineral constituents within the siltstone matrix, generating internal tensile stresses and thermally induced microcracks; differential thermal expansion between mineral grains further causes inter-granular micro-fracturing during repeated heating–cooling cycles. The cumulative effect is a significant increase in microcrack density, directly reducing the cohesion and friction angle of the rock mass.

Under these challenging conditions, the roadway exhibited severe instability. Observed failure modes included large-scale roof collapse and laminar delamination at the roof and rib corners (Fig. 2). Borehole observations revealed a highly fractured zone extending 3-5 m into the ribs and up to 4 m into the roof, characterized by dense oblique and circumferential cracks. The asymmetric nature of the collapse indicates that the failure mechanism is governed by the interaction between the asymmetric stress field and the degraded mechanical properties of the thermally damaged rock mass.

### Laboratory investigation of mechanical degradation

A laboratory testing program was conducted to quantify the mechanical degradation caused by thermal alteration and to identify the underlying mechanisms of the observed roadway instability. Two sets of standard cylindrical specimens (*Φ*50 mm × 100 mm) were prepared for comparison. The first set, unaltered siltstone (US), was collected from the intact siltstone stratum of the No. 5-3 seam overburden at locations confirmed to be beyond the thermal influence boundary, where no visual or mechanical evidence of thermal alteration was observed. The second set, thermally altered siltstone (TAS), was collected from the burnt rock zone directly exposed during roadway excavation. Both specimen types originate from the same stratigraphic horizon at similar depths, ensuring lithological consistency and satisfying the principle of controlled comparison.

**Fig. 3:**
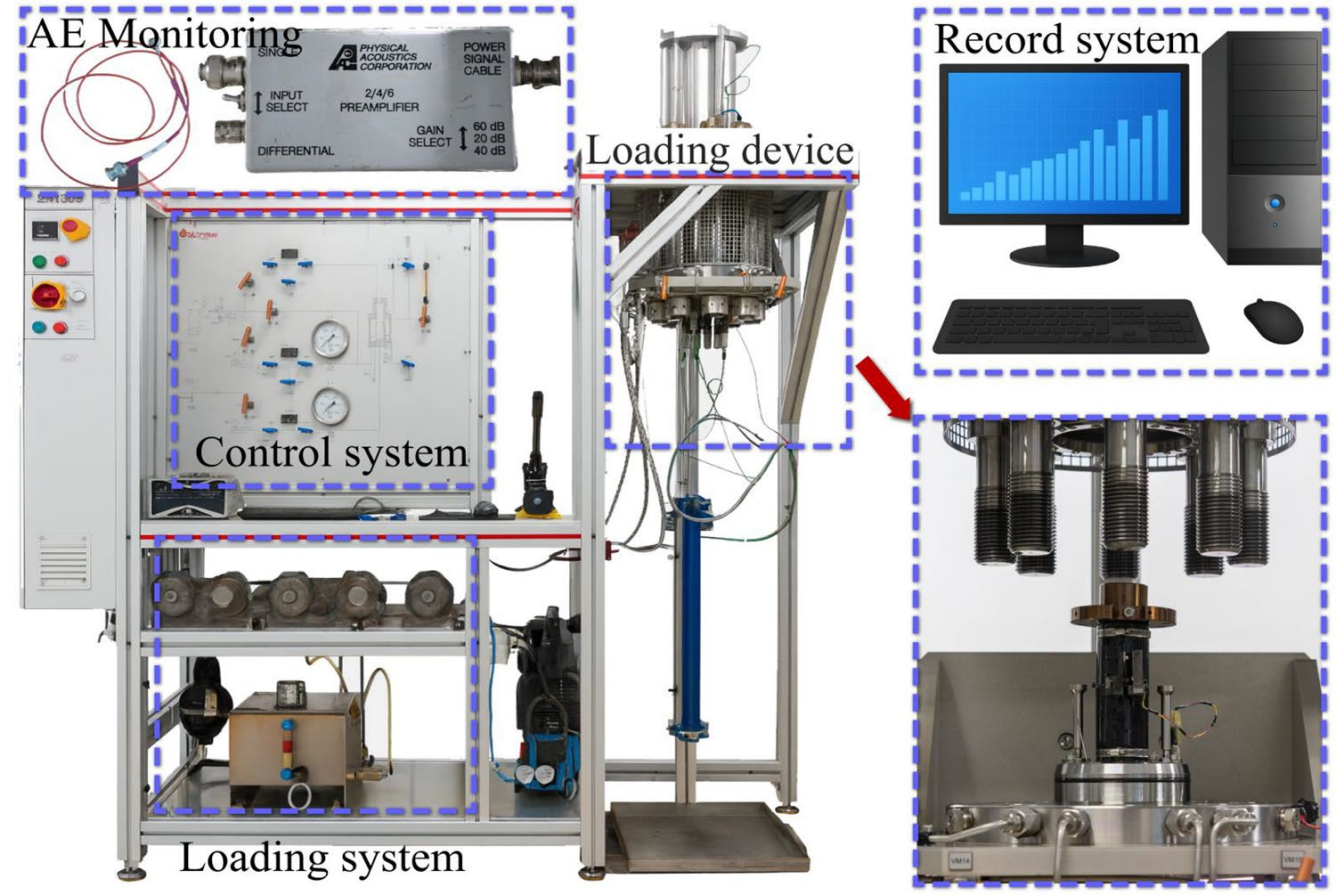
Schematic diagram of the experimental system.

Triaxial compression tests were conducted using a TOP Industrie Rock 600-50 servo-hydraulic rheological testing system. This system is capable of applying a maximum axial stress of 375 MPa and a maximum confining pressure of 60 MPa, with a pressure control accuracy of 0.01 MPa. To simulate the in-situ stress environment, all triaxial compression tests were performed under a constant confining pressure of 10 MPa. Acoustic Emission (AE) monitoring was employed during the triaxial compression to capture the internal micro-fracturing processes underlying the macroscopic mechanical response. AE activity was monitored using a sensor mounted at the base of the triaxial cell. AE signals were amplified by 40 dB, and a data acquisition threshold of 35 dB was used. A schematic of the experimental system is shown in Fig. 3.

**Fig. 4:**
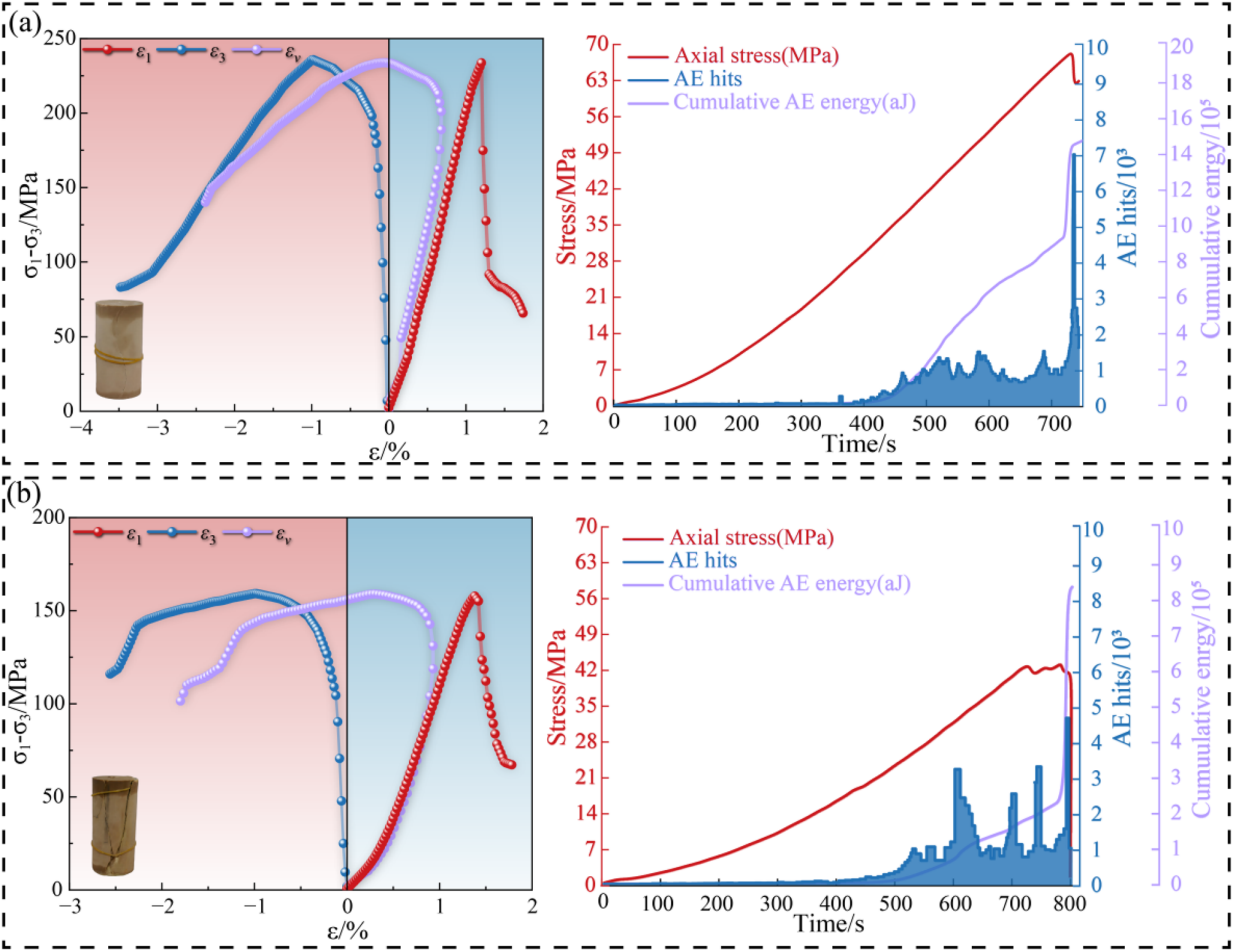
Comparative triaxial compression and AE results. (**a**) US; (**b**) TAS.

The comparative mechanical responses of the US and TAS specimens are shown in Fig. 4. The results indicate a significant degradation of the rock’s mechanical properties due to thermal alteration.

The US specimen (Fig. 4a) exhibits brittle failure characteristics. The stress–strain curve shows a linear elastic phase, reaching a peak deviatoric stress of approximately 240 MPa, followed by an abrupt post-peak stress drop. The failed specimen displays a distinct macroscopic shear fracture. The volumetric strain (*ε*_*v*_) curve shows initial compaction followed by significant dilation near the peak stress, which is characteristic of brittle rock failure. In contrast, the TAS specimen (Fig. 4b) shows a degraded mechanical response. The peak deviatoric stress is reduced to approximately 155 MPa, a 35% decrease compared to the US specimen. The slope of the stress–strain curve is lower, indicating reduced stiffness. The post-peak behavior is characterized by gradual strain-softening. The volumetric strain curve exhibits a prolonged compaction phase and suppressed dilation, suggesting that failure is dominated by the closure of pre-existing microcracks and voids.

The AE monitoring data reveal different internal damage mechanisms. For the US specimen, AE activity was minimal during the elastic loading phase and then increased sharply at peak stress, with a cumulative energy release of 15 × 10^5^ aJ. This pattern corresponds to the rapid formation and coalescence of new microcracks. Conversely, the TAS specimen exhibited continuous AE activity from the onset of loading. The cumulative energy release was 8.5 × 10^5^ aJ, a 43% reduction compared to the US specimen.

These laboratory results are consistent with the field observations (Fig. 2). The findings confirm that the roadway instability is a direct consequence of the mechanical property degradation of the surrounding rock mass caused by long-term thermal exposure.

## Analytical modeling of stress distribution under principal stress rotation

**Fig. 5:**
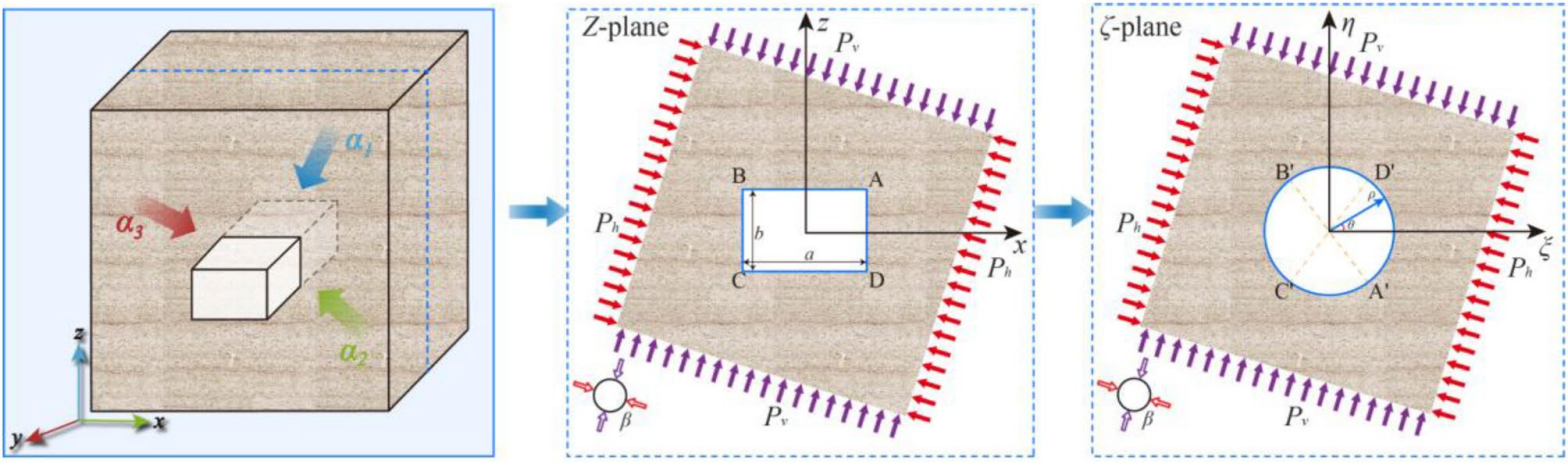
Mechanical model of the rectangular roadway and far-field stress conditions.

Field observations and laboratory results indicate that the observed roadway instability is governed by the interaction between an asymmetric in-situ stress field and the degraded mechanical properties of the thermally damaged rock. To investigate this coupled mechanism, this section develops an analytical model of the stress distribution based on the complex variable method. A Cartesian coordinate system (*x*, *y*, *z*) is defined where the *y*-axis aligns with the longitudinal roadway axis. The analysis is conducted in the transverse *x*–*z* plane, represented as the complex *Z*-plane. Figure 5 illustrates the mechanical model and boundary conditions.

The far-field in-situ stress state is defined by the principal stresses (*σ*_1_, *σ*_2_ *σ*_3_) Considering the arbitrary orientation of the roadway relative to the stress field, the stress components acting on the excavation boundary are determined through tensor transformation^31^:

1 $$\begin{gathered} \sigma_{x}^{\prime } = \cos^{2} \alpha_{1} \cdot \sin^{2} \beta_{1} \cdot \sigma_{1} + \cos^{2} \alpha_{2} \cdot \sin^{2} \beta_{2} \cdot \sigma_{2} + \cos^{2} \alpha_{3} \cdot \sin^{2} \beta_{3} \cdot \sigma_{3} \hfill \\ \sigma_{y}^{\prime } = \cos^{2} \alpha_{1} \cdot \cos^{2} \beta_{1} \cdot \sigma_{1} + \cos^{2} \alpha_{2} \cdot \cos^{2} \beta_{2} \cdot \sigma_{2} + \cos^{2} \alpha_{3} \cdot \cos^{2} \beta_{3} \cdot \sigma_{3} \hfill \\ \sigma_{z}^{\prime } = \sin^{2} \alpha_{1} \cdot \sigma_{1} + \sin^{2} \alpha_{2} \cdot \sigma_{2} + \sin^{2} \alpha_{3} \cdot \sigma_{3} \hfill \\ \tau_{xy}^{\prime } = \cos^{2} \alpha_{1} \cdot \sin \beta_{1} \cdot \cos \beta_{1} \cdot \sigma_{1} + \cos^{2} \alpha_{2} \cdot \sin \beta_{2} \cdot \cos \beta_{2} \cdot \sigma_{2} + \cos^{2} \alpha_{3} \cdot \sin \beta_{3} \cdot \cos \beta_{3} \cdot \sigma_{3} \hfill \\ \tau_{yz}^{\prime } = \cos \alpha_{1} \cdot \sin \beta_{1} \cdot \sin \alpha_{1} \cdot \sigma_{1} + \cos \alpha_{2} \cdot \sin \beta_{2} \cdot \sin \alpha_{2} \cdot \sigma_{2} + \cos \alpha_{3} \cdot \sin \beta_{3} \cdot \sin \alpha_{3} \cdot \sigma_{3} \hfill \\ \tau_{zx}^{\prime } = \cos \alpha_{1} \cdot \cos \beta_{1} \cdot \sin \alpha_{1} \cdot \sigma_{1} + \cos \alpha_{2} \cdot \cos \beta_{2} \cdot \sin \alpha_{2} \cdot \sigma_{2} + \cos \alpha_{3} \cdot \cos \beta_{3} \cdot \sin \alpha_{3} \cdot \sigma_{3} \hfill \\ \end{gathered}$$

For a long roadway, plane strain conditions can be assumed, which simplifies the three-dimensional problem into two independent models. The stability of the roadway, including shear failure at the corners and the development of the plastic zone, is primarily controlled by the in-plane stresses. Therefore, this analysis focuses on the in-plane strain model.

Based on the in-situ measurements the intermediate principal stress the intermediate principal stress(*σ*_2_) is assumed coaxial with the roadway axis(*y*) The major principal stress, *σ*_1_ = *P*_*v*_, and the minor principal stress, *σ*_3_ = *λP*_*v*_, act within the transverse *x*–*z* plane, rotated by an angle *β* relative to the vertical axis. The resulting far-field stress boundary conditions are expressed as:

2 $$\begin{gathered} \sigma_{{x^{\prime } }} = \frac{{P_{v} (1 + \lambda )}}{2} + \frac{{P_{v} (1 - \lambda )}}{2}\cos (2\beta ) \hfill \\ \sigma_{{z^{\prime } }} = \frac{{P_{v} (1 + \lambda )}}{2} - \frac{{P_{v} (1 - \lambda )}}{2}\cos (2\beta ) \hfill \\ \tau_{{zx^{\prime } }} = \frac{{P_{v} (1 - \lambda )}}{2}\sin (2\beta ) \hfill \\ \end{gathered}$$

These expressions directly relate the analytical boundary conditions to field-measurable parameters: the vertical stress magnitude* P*_v_, the lateral pressure coefficient *λ*, and the principal stress rotation angle *β*.

The complex variable method solves this problem by conformally mapping the region outside the rectangular roadway in the *Z*-plane to the region inside a unit circle in the *ζ*-plane. The mapping function is given by:

3 $$z = \omega (\zeta ) = R(\frac{1}{\zeta } + c_{1} \zeta + c_{3} \zeta^{3} )$$

The mapping parameters, *R* and the shape constant *k*, are determined as functions of the roadway height (*a*) and width (*b*) by solving the following system of equations derived from two symmetry points on the roadway boundary:

4 $$\left\{ \begin{gathered} R = \frac{a}{{1 + c_{1} + c_{3} }} = \frac{a}{{1 + \cos (2k\pi ) - \frac{1}{6}\sin^{2} (2k\pi )}} \hfill \\ k = \frac{1}{2\pi }\arccos \left( {\frac{{3a + 3b - 2\sqrt {a^{2} + 7ab + b^{2} } }}{a - b}} \right) \hfill \\ \end{gathered} \right.$$

According to Muskhelishvili’s theory^32^, the stress field for a plane strain problem is determined by two analytic potential functions, *φ*(*ζ*) and *ψ*(*ζ*), with the general forms:

5 $$\left\{ {\begin{array}{*{20}l} {\phi (\zeta ) = B \cdot \omega (\zeta ) + \phi_{0} (\zeta )} \hfill \\ {\psi (\zeta ) = (B^{\prime } + iC^{\prime } )\omega (\zeta ) + \psi_{0} (\zeta )} \hfill \\ \end{array} } \right.$$

The coefficients *B*, *B*′, and *C*′ are governed by the far-field stress state and the principal stress rotation angle *β*:

6 $$\left\{ \begin{gathered} B = \frac{1}{4}(\sigma_{{x^{\prime}}} + \sigma_{{z^{\prime}}} ) = \frac{{P_{v} (1 + \lambda )}}{4} \hfill \\ B^{\prime } = \frac{1}{2}(\sigma_{{z^{\prime}}} - \sigma_{{x^{\prime } }} ) = - \frac{{P_{v} (1 - \lambda )}}{2}\cos (2\beta ) \hfill \\ C^{\prime } = \tau_{{zx^{\prime } }} = \frac{{P_{v} (1 - \lambda )}}{2}\sin (2\beta ) \hfill \\ \end{gathered} \right.$$

By applying the boundary conditions at the excavation surface, the complete expressions for *φ*_0_(ζ), *ψ*_0_(ζ), *φ*(ζ) and *ψ*(ζ) are derived through complex variable calculations:

7 $$\begin{gathered} \phi_{0} (\zeta ) = \left( { - \frac{{R[(P_{v} + \lambda P_{v} )c_{1} + (P_{v} - \lambda P_{v} )\cos 2\beta ]}}{{2(1 - c_{3} )}} - i\frac{{R(P_{v} - \lambda P_{v} )\sin 2\beta }}{{2(1 + c_{3} )}}} \right)\zeta - 2BRc_{3} \zeta^{3} \hfill \\ \psi_{0} (\zeta ) = - \frac{R}{2}\left[ {(P_{v} + \lambda P) + (P_{v} - \lambda P_{v} )c_{1} e^{ - 2i\beta } } \right]\zeta - \frac{{Rc_{3} (P_{v} - \lambda P_{v} )}}{2}e^{ - 2i\beta } \zeta^{3} - \frac{{\overline{{a_{1} }} c_{3} }}{\zeta } - \frac{{R\left( {\frac{1}{\zeta } + c_{1} \overline{\zeta } + c_{3} \overline{\zeta }^{3} } \right)}}{{R\left( { - \frac{1}{{\zeta^{2} }} + c_{1} + 3c_{3} \zeta^{2} } \right)}}(a_{1} + 3a_{3} \zeta^{2} ) \hfill \\ \end{gathered}$$

8 $$\begin{gathered} \phi (\zeta ) = \frac{{P_{v} + \lambda P_{v} }}{4\zeta }R + (\frac{{P_{v} + \lambda P_{v} }}{4}Rc_{1} + a_{1} )\zeta + (\frac{{P_{v} + \lambda P_{v} }}{4}Rc_{3} + a_{3} )\zeta^{3} \hfill \\ \psi (\zeta ) = - (\frac{{P_{v} - \lambda P_{v} }}{2}\cos 2\beta + i\frac{{P_{v} - \lambda P}}{2}\sin 2\beta )\left( {\frac{R}{\zeta } + Rc_{1} \zeta + Rc_{3} \zeta^{3} } \right) + \psi_{0} (\zeta ) \hfill \\ \end{gathered}$$

The stress components in curvilinear coordinate system (*ρ*, *θ*) of the *ζ*-plane are obtained:

9 $$\left\{ \begin{gathered} \sigma_{\rho } + \sigma_{\theta } = 4{\mathrm{Re}} \left[ {\frac{{\phi^{\prime } (\zeta )}}{{\omega^{\prime } (\zeta )}}} \right] \hfill \\ \sigma_{\theta } - \sigma_{\rho } + 2i\tau_{\rho \theta } = \frac{{2\zeta^{2} }}{{\rho^{2} \omega^{\prime } (\zeta )}}\left[ {\frac{{\overline{\omega (\zeta )} }}{\omega (\zeta )}\left( {\frac{{\phi^{\prime } (\zeta )}}{{\omega^{\prime } (\zeta )}}} \right)^{\prime } + \psi^{\prime } (\zeta )} \right] \hfill \\ \end{gathered} \right.$$

Finally, to facilitate failure analysis, these components are transformed back to the Cartesian coordinate system (*x*, *z*) using a transformation angle *α*:

10 $$\alpha = \arctan (\frac{{(c_{1} \rho + \frac{1}{\rho })\sin \theta + 3c_{3} \rho^{3} \sin (3\theta )}}{{(c_{1} \rho - \frac{1}{\rho })\cos \theta + 3c_{3} \rho^{3} \cos (3\theta )}})$$

11 $$\begin{gathered} \sigma_{x} = \sigma_{\rho } \sin^{2} \alpha + \sigma_{\theta } \cos^{2} \alpha - \tau_{\rho \theta } \sin (2\alpha ) \hfill \\ \sigma_{z} = \sigma_{\rho } \cos^{2} \alpha + \sigma_{\theta } \sin^{2} \alpha + \tau_{\rho \theta } \sin (2\alpha ) \hfill \\ \tau_{xz} = \frac{1}{2}(\sigma_{\rho } - \sigma_{\theta } )\sin (2\alpha ) + \tau_{\rho \theta } \cos (2\alpha ) \hfill \\ \end{gathered}$$

This analytical solution provides a quantitative description of the elastic stress distribution around a rectangular roadway as a function of stress field parameters (*P*_v_, *λ*, *β*) and the roadway geometry (*a*, *b*). It serves as the theoretical basis for the subsequent validation and parametric study of the failure mechanism.

It should be noted that the solution is based on several inherent assumptions. The linear elastic and homogeneous assumption means that stress predictions near the plastic zone may overestimate actual values in highly fractured rock masses. The equivalent circular approximation for plastic zone estimation captures the macroscopic extent reliably but may not reproduce detailed local geometry under strongly asymmetric loading. The plane strain assumption is appropriate for long roadway cross-sections but should be applied with caution near working faces where three-dimensional effects are significant.

## Analysis of the roadway failure mechanism

This section validates the analytical solution and subsequently conducts a parametric analysis to investigate the coupled effects of the in-situ stress field and rock mass properties on the roadway failure mechanism.

### Validation of the analytical model

To verify the accuracy of the derived analytical solution, the results were benchmarked against numerical simulations performed using FLAC3D. The numerical model dimensions were 100 m × 1 m × 100 m, containing a rectangular roadway of 5.2 m in height and 3.0 m in width. The rock mass was modeled as an elastic material with modulus *E* = 3.1 GPa and a Poisson’s ratio *µ* = 0.25, with a vertical stress of* P*_v_=10 MPa.

**Fig. 6:**
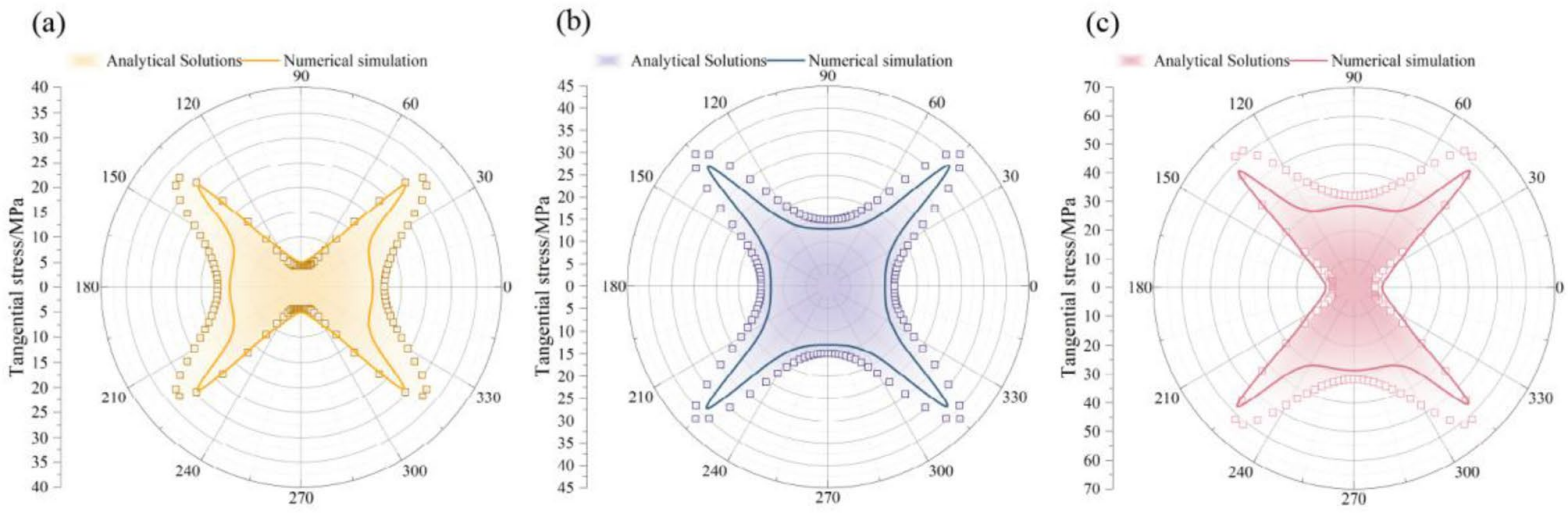
Comparison of analytical and numerical stress distributions. (**a**) *λ* = 1.0, (**b**) *λ* = 1.5, (**c**) *λ* = 2.0.

Figure 6 compares the analytical and numerical results for three lateral pressure coefficients: *λ* = 0.5, 1.0, and 2.0. In all cases, the stress distribution patterns and magnitudes from the analytical solution are in close agreement with the numerical results. This comparison validates the accuracy of the analytical solution for representing the elastic stress field around the rectangular roadway.

The analytical solution is also used to estimate the extent of the plastic zone. The approach is based on a semi-analytical method where the maximum tangential stress (*σ*_θ,max_) at the excavation boundary, calculated from the elastic solution, is used in conjunction with an equivalent circular roadway model. This maximum stress is defined as:

12 $$\sigma_{\theta ,\max } = \max_{\theta \in [0,2\pi ]} \left\{ {\sigma_{\theta } (\theta )} \right\} = \max_{\theta \in [0,2\pi ]} \left\{ {4{\mathrm{Re}} \left[ {\frac{{\phi^{\prime } \left( {e^{i\theta } } \right)}}{{\omega^{\prime } \left( {e^{i\theta } } \right)}}} \right] - p_{i} } \right\}$$

This stress concentration is then used to estimate the radius of the plastic zone (*R*_p_) as:

13 $$R_{p} = R_{{{\mathrm{eq}}}} \left[ {\frac{{\left( {\sigma_{\theta ,\max } + p_{i} } \right)(\xi - 1) + 2\sigma_{c} }}{{\left( {p_{i} \left( {\xi - 1} \right) + \sigma_{c} } \right)\left( {\xi + 1} \right)}}} \right]^{{\frac{1}{\xi - 1}}}$$

where, *R*_eq_ is the radius of the equivalent circular roadway, *p*_i_ is the support resistance, and the parameters *σ*_c_ and *ξ* are strength-related parameters.

**Fig. 7:**
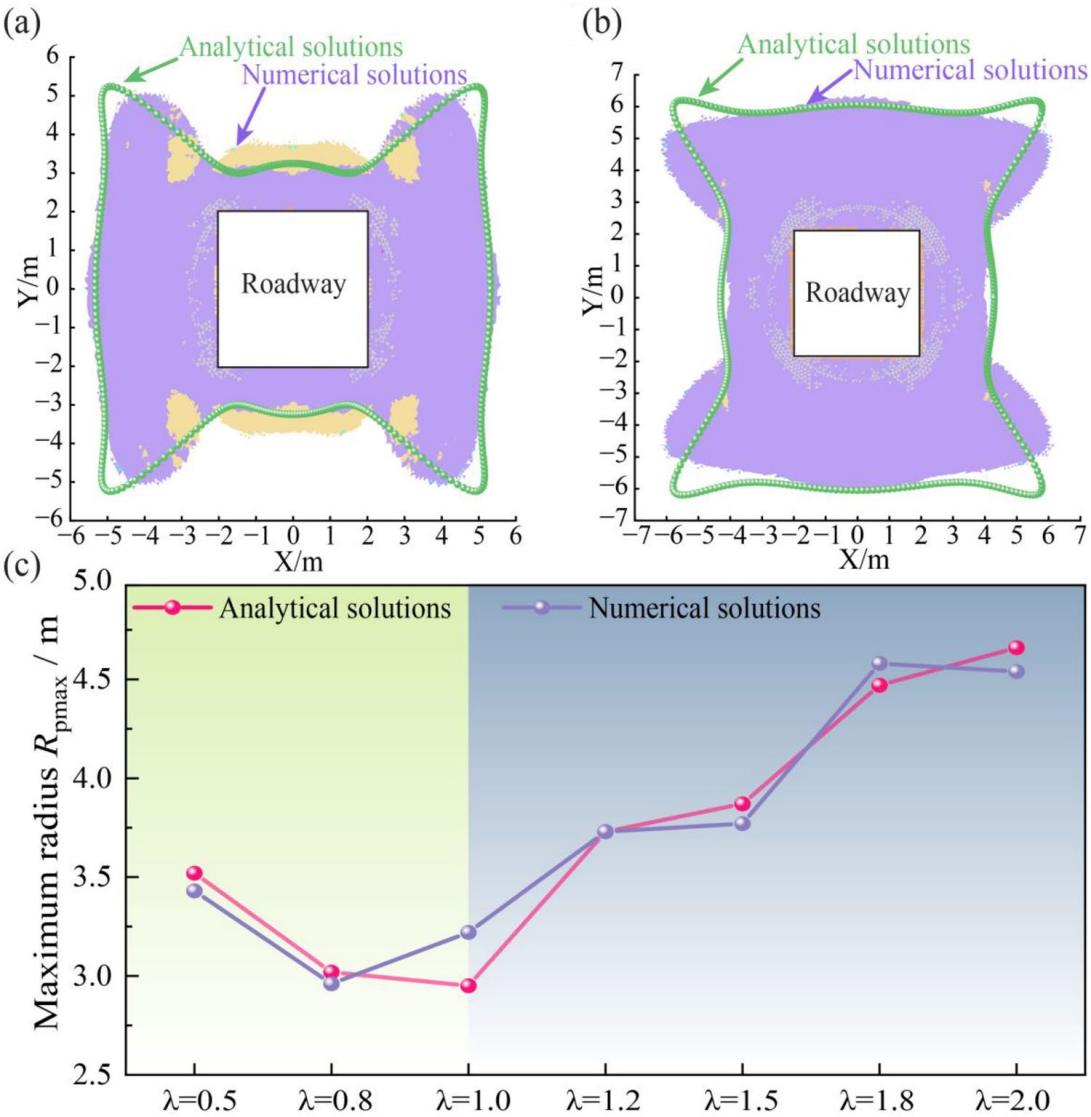
Comparison of the plastic zone under different *λ*. (**a**) *λ* = 0.5, (**b**) *λ* = 2.0, (**c**) *R*_pmax_.

The estimated plastic zone morphologies for λ = 0.5 and 2.0 are presented in Fig. 7. The plastic zone morphology and maximum extent (*R*_pmax_) predicted by the semi-analytical approach correspond well with the numerical simulations. Although minor deviations exist, the method accurately captures the macroscopic evolution of the plastic zone as the stress ratio varies.

### Parametric analysis of controlling factors

**Fig. 8:**
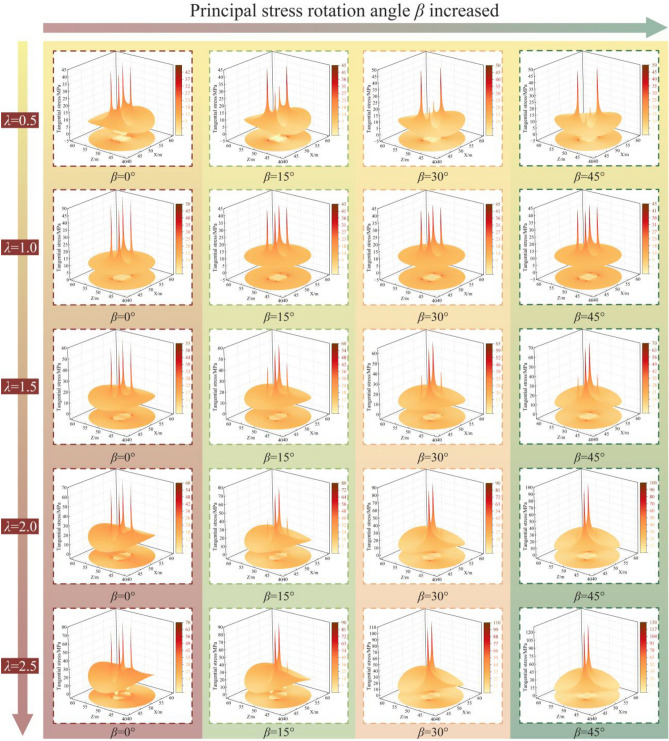
Evolution of tangential stress distribution under varying *λ* and *β*.

This analysis investigates how external stress parameters (λ, β) and intrinsic rock mass properties (C, φ) govern the stress distribution and plastic zone evolution. Figure 8 illustrates the distribution of tangential stress at the roadway boundary under various combinations of *λ* and* β*.

The results indicate that the principal stress rotation angle *β* is a primary factor controlling stress asymmetry. As *β* increases from 0° to 45°, the locations of peak tangential stress migrate from the midpoints of the ribs and roof toward one pair of diagonally opposite corners. This rotation creates zones of high stress concentration at these corners, forming dominant stress zones, while the opposing corners experience stress relief, forming subordinate stress zones.

The lateral pressure coefficient (*λ*) magnifies this asymmetric distribution. For a given rotation angle (*β* = 30°), increasing λ from 0.5 to 2.5 results in a substantial, non-linear increase in the peak tangential stress. This demonstrates that a high lateral pressure coefficient not only elevates the overall stress magnitude but also accentuates the non-uniformity of the stress distribution around the roadway.

**Fig. 9:**
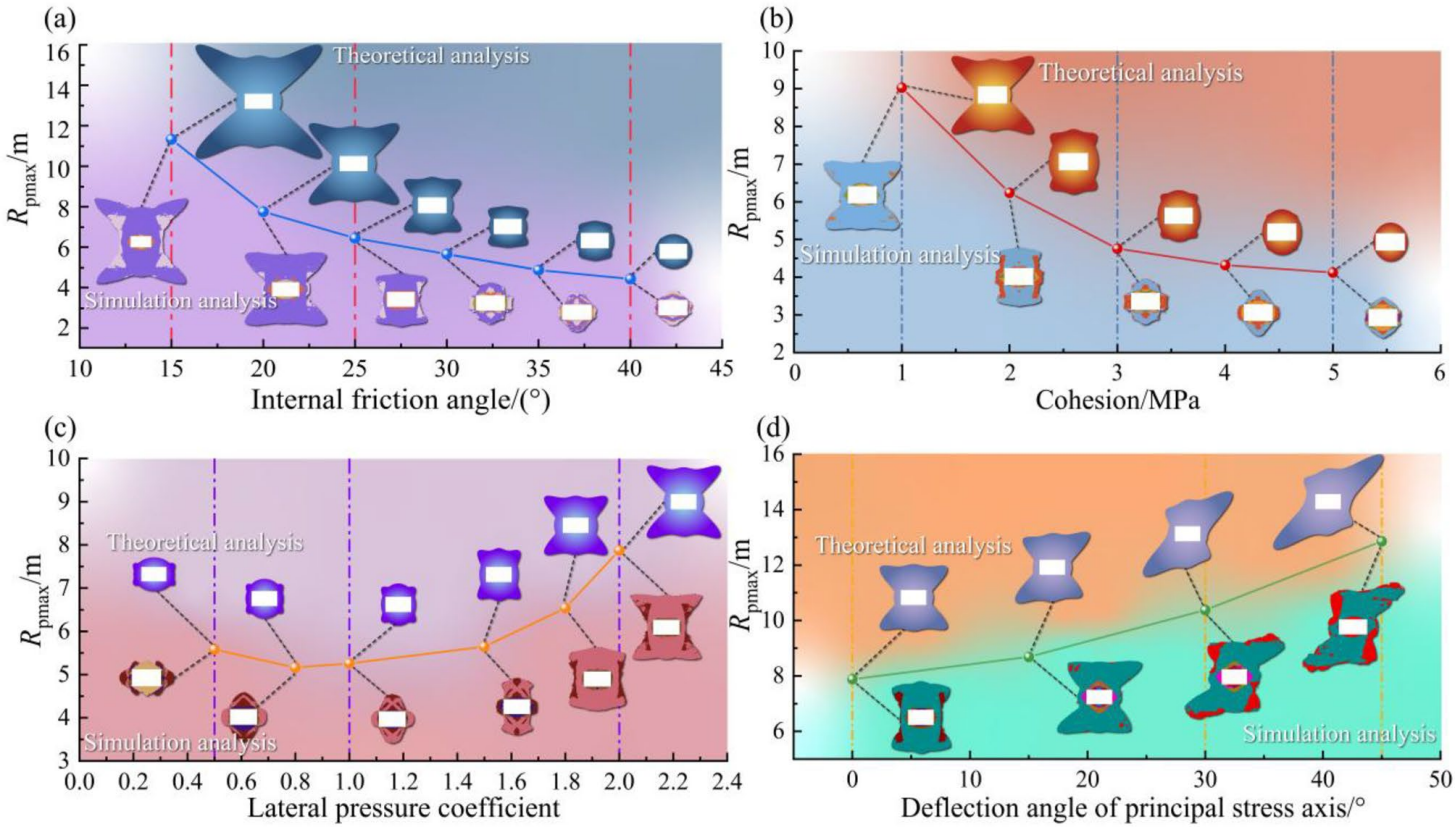
Sensitivity analysis of plastic zone extent *R*_pmax_ to key parameters. (**a**) *φ*, (**b**) *C*, (**c**) *λ*, (**d**) *β*.

The evolution of the plastic zone results from the interaction between the stress field and the rock mass strength. The influence of external stress parameters (*λ*, *β*) and intrinsic rock mass strength parameters (*C*, *φ*) on the shape and extent of the plastic zone is presented in Fig. 9.

As shown in Fig. 9a, b, *R*_pmax_ decreases non-linearly as either the* φ* increases from 15° to 40° or *C* increases from 1 to 5 MPa. This effect is most pronounced in lower-strength rock masses (*C* < 3 MPa,* φ* < 30°), where *R*_pmax_ decreases from 11 to 5 m. For higher strength rock, the rate of reduction is less pronounced, with *R*_pmax_ decreasing from 5 m to 4.2 m. This is because higher *C* and φ values increase the shear strength of the rock mass, enhancing its capacity to resist excavation-induced stresses.

The stress field characteristics also exert significant control, as illustrated in Fig. 9c, d The relationship between *R*_pmax_ and *λ* is non-monotonic and follows a U-shaped trend. *R*_pmax_ decreases from 5.5 m at *λ* = 0.5 to a minimum of 3.5 m under *λ* = 1.0 conditions, before increasing to 7.8 m at *λ* = 2.0. The β influences both the size and the shape of the plastic zone. *R*_pmax_ increases from 7.8 m at *β* = 0° to 12.5 m at *β* = 45°. Concurrently, the plastic zone geometry transforms from a symmetric distribution to an asymmetric, inclined X-shaped pattern. This transformation is a direct result of stress redistribution, which concentrates stress at diagonal corners and guides failure propagation along preferential paths.

### The coupled failure mechanism and quantitative contribution analysis

The analysis reveals a coupled failure mechanism governing roadway instability. The external stress field dictates the asymmetric geometry of the failure. Specifically, the principal stress rotation (*β*) concentrates stress at diagonally opposite corners, defining initiation points and guiding damage propagation. This process results in the characteristic inclined X-shaped plastic zone. High lateral pressure (*λ*) further magnifies these concentrations, expanding the failure envelope. Concurrently, the intrinsic strength of the rock mass governs the failure scale. Laboratory tests confirmed that thermally damaged rock exhibits significantly reduced cohesion (*C*) and internal friction angle (*φ*). The parametric study demonstrates that this degraded capacity to resist shear stress leads to the development of an extensive plastic zone.

To quantify the relative contributions of the two governing factors, a four-case decoupling analysis was conducted using the analytical model. The four cases are defined in Table 1 below.

**Table 1:** Decoupling analysis of stress asymmetry and thermal damage contributions.

Case	Stress condition	Rock properties	*R*_*pmax*_ (m)	Asymmetry index
C1	Coaxial (*β* = 0°, *λ* = 1.8)	Unaltered (*C* = 4 MPa, *φ* = 30°)	5.5	Symmetric
C2	Rotated (*β* = 15°, *λ* = 1.8)	Unaltered (*C* = 4 MPa, *φ* = 30°)	8.5	Asymmetric
C3	Coaxial (*β* = 0°, *λ* = 1.8)	Thermally damaged (*C* = 2 MPa, *φ* = 20°)	7.5	Symmetric
C4	Rotated (*β* = 15°, *λ* = 1.8)	Thermally damaged (*C* = 2 MPa, *φ* = 20°)	10.5	Asymmetric

The results of the decoupling analysis reveal the distinct and complementary roles of the two governing factors. When principal stress rotation alone is introduced at *β* = 15°, *R*_*pmax*_ increases from 5.5 m to 8.5 m, a rise of 3.0 m or 54.5%, and the plastic zone geometry transforms from symmetric to an asymmetric inclined X-shaped pattern. This demonstrates that the asymmetric stress field primarily controls the geometry and orientation of the failure zone. When thermal degradation alone is considered, with *C* reduced from 4 to 2 MPa and *φ* reduced from 30° to 20°, *R*_*pmax*_ increases from 5.5 m to 7.5 m, a rise of 2.0 m or 36.4%, while the plastic zone geometry remains symmetric. This confirms that mechanical degradation primarily governs the scale and extent of the failure zone. In the coupled scenario, *R*_*pmax*_ reaches 10.5 m, which exceeds the sum of the two individual increments of 3.0 m and 2.0 m. This super-additive effect reflects the physical essence of the coupled failure mechanism: the weakened rock mass is far less capable of resisting the stress concentrations at the dominant corners, so the combined effect of stress asymmetry and thermal degradation produces a disproportionately large and asymmetric failure zone.

In summary, the large-scale and asymmetric failure observed in the field is a direct consequence of this coupled mechanism: an asymmetric stress field acts upon a rock mass with significantly reduced mechanical properties. The identified failure mechanism provides the basis for the targeted support strategy developed in the next section.

## Synergistic control strategy and performance analysis

The preceding analysis identified a coupled failure mechanism responsible for roadway instability. An asymmetric stress field concentrates loads at the roadway corners, and the mechanical degradation of the thermally damaged rock limits its capacity to resist these loads. To address this mechanism, a targeted synergistic control strategy, termed the Reinforcement-Anchorage-Confinement (RAC) system, was developed.

### The reinforcement-anchorage-confinement (RAC) system

**Fig. 10:**
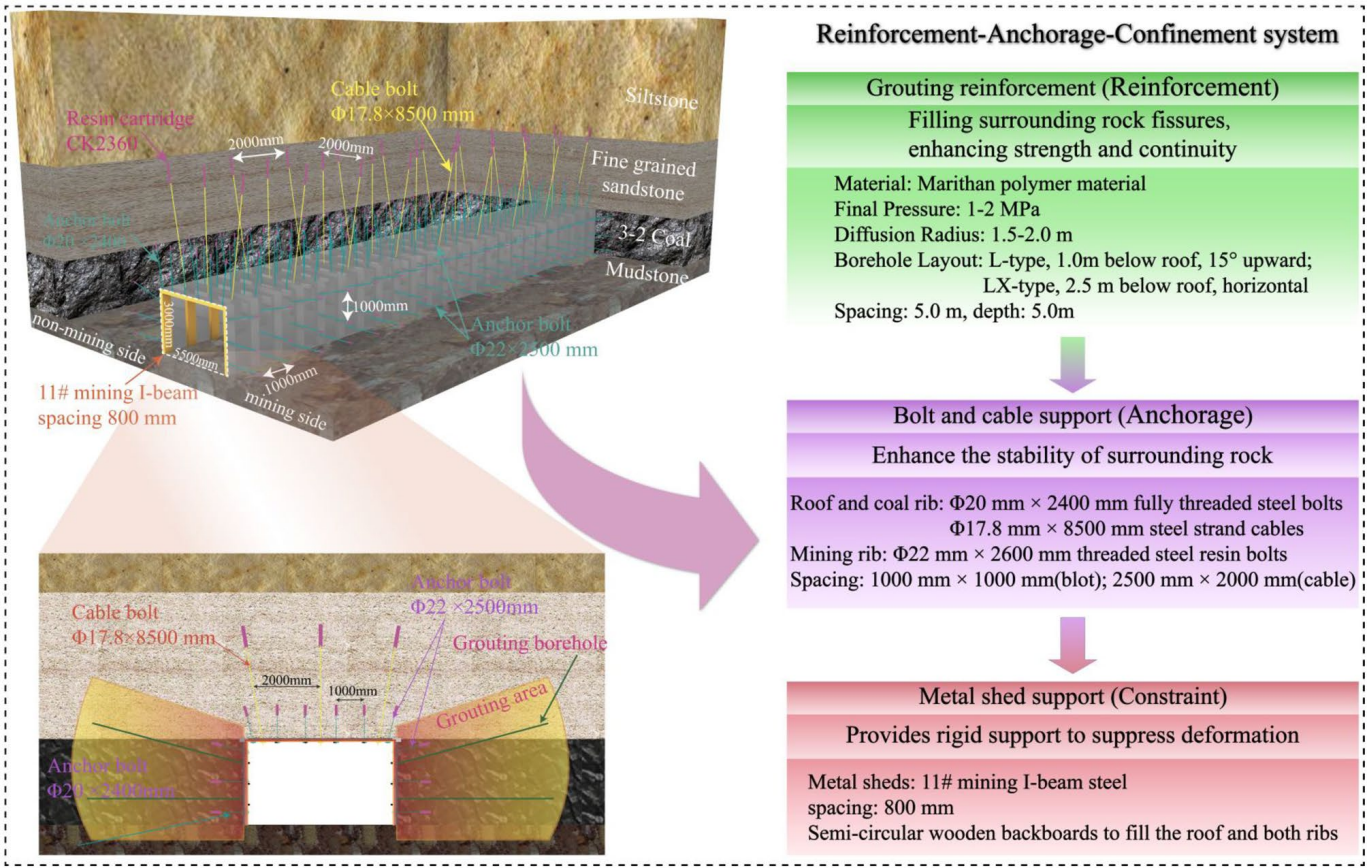
Configuration of the proposed Reinforcement-Anchorage-Confinement (RAC) support system.

The RAC system is a multi-component strategy designed to counteract the identified coupled failure mechanism by integrating three complementary functions: Reinforcement, Anchorage, and Confinement. The comprehensive configuration is illustrated in Fig. 10. The reinforcement component, achieved through grouting, improves the integrity and residual strength of the fractured rock mass. The anchorage component, consisting of high strength rock bolts and cables, provides deep-seated support to mobilize the rock mass’s self-bearing capacity and resist asymmetric stress concentrations. The confinement component, provided by heavy-duty U-steel sets, delivers immediate surface restraint to control large-scale deformation.

### Numerical evaluation of support performance

The performance of the RAC system was evaluated using UDEC, which is particularly suitable for simulating the discontinuous behavior of fractured rock masses. A two-dimensional numerical model with dimensions of 100 m × 100 m was established, centering on a rectangular roadway (5.5 m wide × 3.0 m high). The region surrounding the excavation was discretized into triangular blocks to represent the fractured nature of the rock mass. Boundary conditions were applied consistent with the in-situ stress measurements. The rock mass was simulated using the Mohr–Coulomb constitutive model with properties derived from the laboratory tests.

The support components of the RAC system were simulated using structural elements available in UDEC. The anchorage system (rock bolts and cables) was modeled using Cable elements, while the U-steel sets were represented by Beam elements. The mechanical properties for these components are detailed in Table 2. The reinforcement effect of grouting was simulated by enhancing the joint properties within the designated grouted zone. Based on previous research^33^, the cohesion was increased by 20%, tensile strength by 50 kPa, and friction angle by 5°.

**Table 2:** Mechanical properties of support components in the UDEC model.

Support element	Parameter	Value
Rockbolt	Elastic Modulus/GPa	200
	Yield Force/kN	120
	Bond Stiffness/(GN·m^−2^ )	2
	Bond Strength/(GN·m^−2^ )	0.4
Cable	Elastic Modulus/GPa	200
	Yield Force/kN	269
	Bond Stiffness/(GN·m^−2^ )	2
	Bond Strength/(GN·m^−2^ )	0.4
Beam	Elastic Modulus/GPa	200
	Yield Strength/MPa	500
	Coupled Normal Stiffness/(GPa·m^−1^ )	10
	Coupled Shear Stiffness/(GPa·m^−1^ )	10

#### Analysis of fracture control

**Fig. 11:**
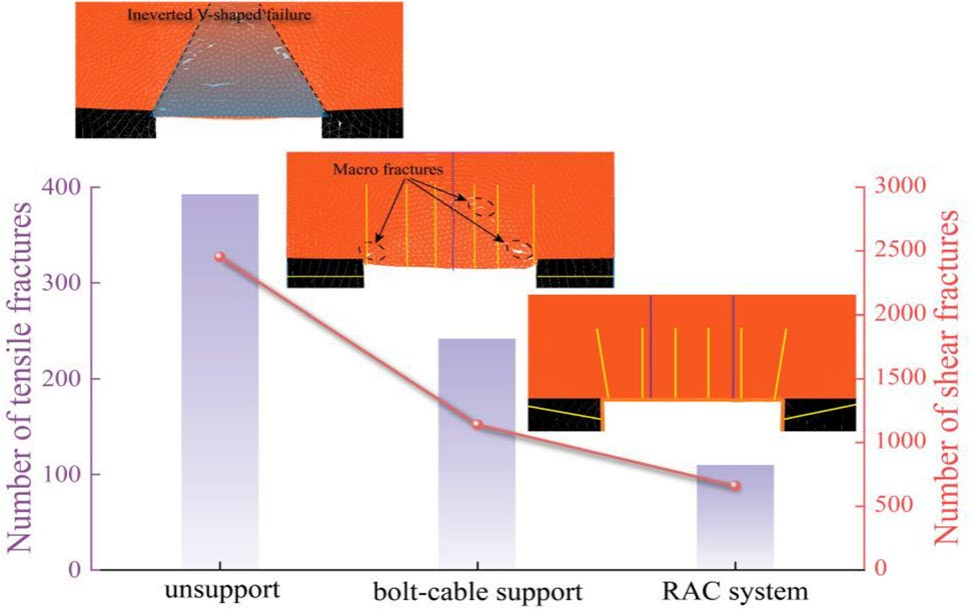
Comparison of roof fracture development under different support conditions.

The effectiveness of different support strategies was evaluated by comparing the simulated fracture development in the roadway roof, as presented in Fig. 11. In the unsupported case, the rock mass exhibited extensive failure, forming a distinct inverted V-shaped shear failure zone in the roof. This shear dominant mechanism corresponds to the large-scale collapse observed in the field. Under the original support condition, an improvement in rock mass stability was observed. The anchorage system provided reinforcement and shear resistance, resulting in a 38.3% reduction in shear cracks and a 53.5% reduction in tensile cracks compared to the unsupported case. However, a deep-seated potential for shear failure remained, indicating that the anchorage system alone could not fully control the extent of the fractured zone.

The implementation of the RAC system resulted in more effective strata control. The integrated approach suppressed the formation of the inverted V-shaped failure zone. Compared to the unsupported case, the total reduction in shear and tensile cracks reached 72.1% and 73.0%, respectively. This demonstrates that the RAC system alters the failure pattern from large-scale, uncontrolled fracturing to localized, contained deformation, which contributes to the long-term stability of the roadway.

#### Optimization of stress environment

**Fig. 12:**
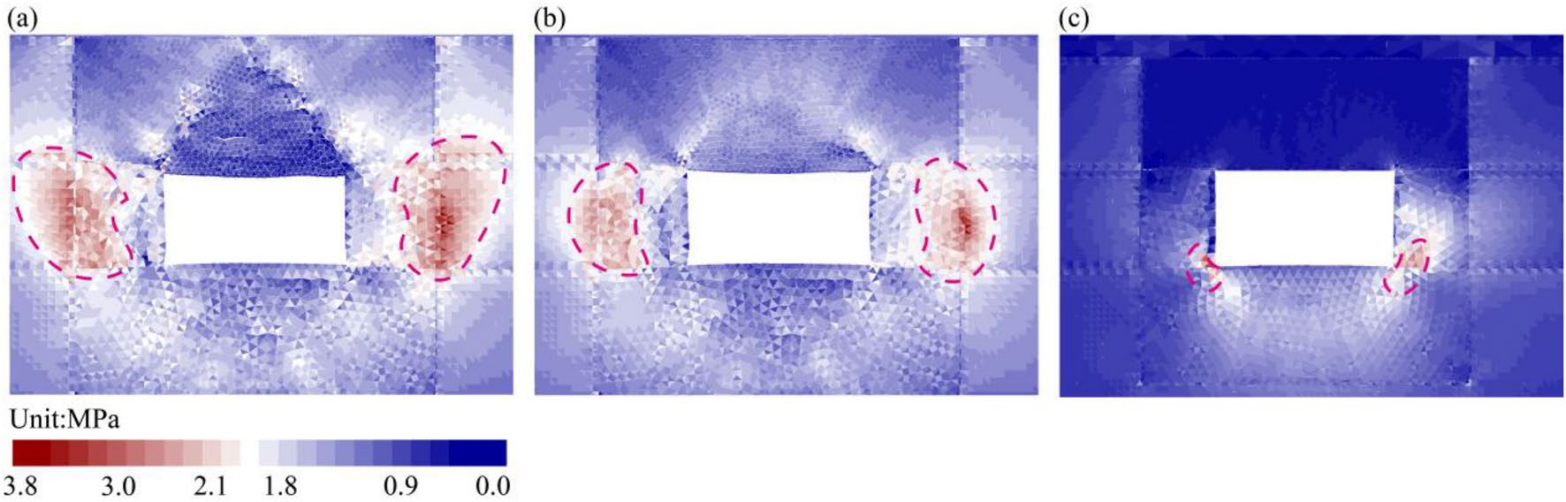
Distribution of deviatoric stress under different support conditions. (**a**) Unsupported; (**b**) Original Support; (**c**) RAC system.

The deviatoric stress is a key indicator of the potential for plastic failure in the rock mass. The distribution of deviatoric stress around the roadway for each support condition was analyzed to evaluate how the support systems altered the stress environment, as shown in Fig. 12.

In the unsupported case (Fig. 12a), a high deviatoric stress zone with a peak value of approximately 3.8 MPa developed at a distance of about 2.6 m into the ribs. The region immediately surrounding the excavation exhibited low deviatoric stress, suggesting that this shallow rock had yielded and transferred stress deeper into the surrounding rock. Under the original support condition (Fig. 12b), the anchorage system stabilized the shallow rock. Consequently, the peak deviatoric stress zone moved closer to the roadway surface to a distance of approximately 1.4 m. However, a considerable surrounding rock remained subjected to high deviatoric stress.

The application of the RAC system (Fig. 12c) resulted in a significant alteration of the stress distribution. The extensive high-stress zones within the ribs were suppressed. Instead, the deviatoric stress became concentrated in small, contained zones at the floor corners, with a peak value of approximately 3.7 MPa. This indicates that the RAC system forms a competent load-bearing structure around the roadway. This reinforced zone carries the abutment stresses, preventing stress transfer into the surrounding rock and thereby reducing the rock susceptible to yielding.

#### Mechanical response of support components

**Fig. 13:**
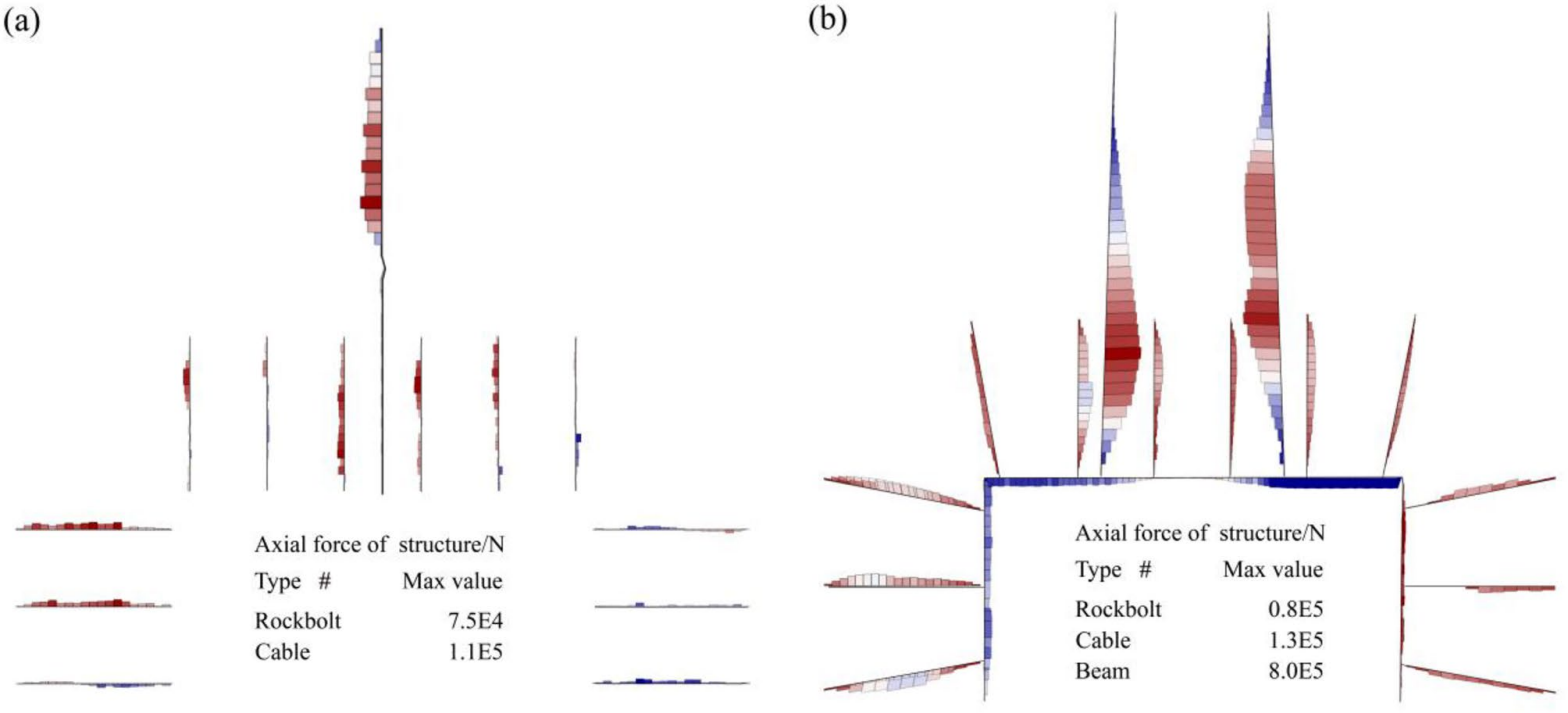
Axial force distribution: (**a**) bolt-cable support; (**b**) RAC system.

The axial forces developed in the support components were analyzed to evaluate the load-sharing mechanism under each support condition. The results are presented in Fig. 13.

Under the original support condition (Fig. 13a), the anchorage system exhibited low load utilization. The maximum axial force in the rock bolts was 82 kN, representing only 28% of the bolt’s breaking load. Similarly, the maximum axial force in the cables was 140 kN, just 18% of the cable’s breaking load. This low utilization occurs because shear failure in the deep rock mass compromises the anchorage zones of the bolts and cables. This damage prevents the support elements from developing their full load-bearing capacity and compromises their effectiveness.

In contrast, the application of the RAC system resulted in a synergistic interaction between the support components (Fig. 13b). The average axial force in the rock bolts increased to 58 kN (48% of yield load), and the average axial force in the cables increased to 84 kN (32% of yield load), indicating a significant improvement in utilization. The steel beams provided immediate surface confinement and carried a substantial load, with a maximum axial force reaching 800 kN. The analysis shows that the high stiffness confinement (steel sheds) and reinforcement (grouting) components work in concert to stabilize the rock mass. This stabilization creates a favorable stress environment, allowing the anchorage (bolts and cables) elements to become effectively engaged. This efficient, synergistic load-sharing is fundamental to the performance of the RAC system in controlling roadway stability in thermally damaged rock.

## Field application and performance verification

**Fig. 14:**
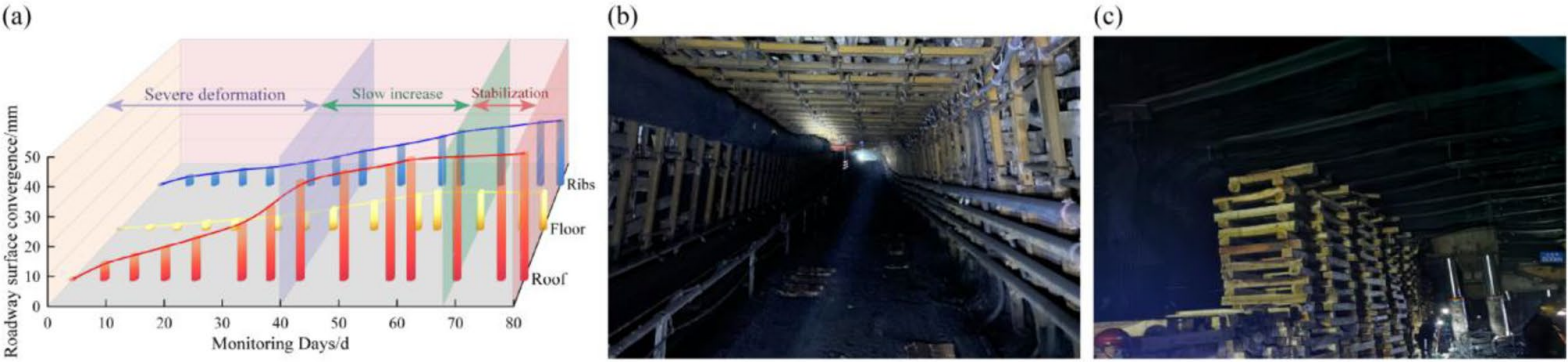
Field performance of the RAC system. (**a**) Monitoring data, (**b**) Final support effect, (**c**) Advance roadway of working face.

To validate the performance of the proposed RAC system under in-situ conditions, the system was implemented in a test section of the 53122 working face return airway. The performance of the system was systematically evaluated by monitoring roadway convergence. The results are presented in Fig. 14.

The monitored deformation data, shown in Fig. 14a, indicate three distinct stages. During the initial 40 days following installation, the roadway exhibited primary deformation as the support system and the surrounding rock mass adjusted to the redistributed stresses. From 40 to 68 days, the rate of convergence decreased substantially, indicating the formation of a stable, load-bearing structure. After 68 days, the deformation rate became negligible, and the roadway reached a state of equilibrium. The final measured values for maximum roof subsidence and maximum rib convergence were 37 mm and 19 mm, respectively. These displacement values are well within the mine’s operational limits, which confirms that the large-scale instability was effectively controlled.

The roadway remained stable, with no visible evidence of significant new fracturing or roof collapse (Fig. 14b, c). The field performance validates the design principles of the RAC system. By integrating reinforcement to improve rock mass integrity, anchorage to resist asymmetric stresses, and confinement to control surface deformation, the system successfully stabilized the roadway in the weak, thermally damaged rock mass.

## Conclusion

This study investigated the stability mechanisms and control of roadways excavated in thermally damaged rock masses under asymmetric stress. Based on an integrated approach of laboratory testing, theoretical modeling, numerical simulation, and in-situ validation, the following conclusions are drawn:

1. Thermal alteration substantially degrades the mechanical properties of the rock, serving as a primary cause of roadway instability. Laboratory tests confirmed a marked reduction in the strength and stiffness of the thermally damaged rock. Acoustic Emission monitoring indicated that this degradation is attributable to the coalescence of thermally induced microcracks, which shifts the failure process from brittle, energy-storing behavior to a more ductile, progressive damage mode.
2. Roadway failure is governed by a coupled mechanism involving the asymmetric stress field and the degraded rock mass properties. An analytical solution incorporating the effect of principal stress rotation was developed and validated. The analysis demonstrated that principal stress rotation dictates the asymmetric, inclined X-shaped geometry of the failure zone, while the reduced strength of the thermally damaged rock governs the large scale of the resulting instability.
3. The proposed RAC system is an effective control strategy for these conditions, as its design directly addresses the identified coupled failure mechanism. Numerical simulations and a field application verified its performance. The system functions through a synergistic interaction of its components, which alters the failure mode from large-scale fracturing to contained deformation and redistributes stress to form a stable load-bearing structure around the roadway.
4. The field implementation of the RAC system successfully controlled roadway deformation. In-situ monitoring confirmed that final roof subsidence and rib convergence were maintained at 37 mm and 19 mm, respectively, demonstrating the long-term stability of the roadway.

## Data Availability

The datasets used and/or analysed during the current study available from the corresponding author on reasonable request.
